# Estimation of inhalation flow profile using audio-based methods to assess inhaler medication adherence

**DOI:** 10.1371/journal.pone.0191330

**Published:** 2018-01-18

**Authors:** Terence E. Taylor, Helena Lacalle Muls, Richard W. Costello, Richard B. Reilly

**Affiliations:** 1 Trinity Centre for Bioengineering, Trinity College Dublin, The University of Dublin, Dublin, Ireland; 2 School of Engineering, Trinity College Dublin, The University of Dublin, Dublin, Ireland; 3 Faculty of Medicine, Universitat de Barcelona, Barcelona, Spain; 4 Department of Medicine, Royal College of Surgeons in Ireland, Dublin, Ireland; 5 School of Medicine, Trinity College Dublin, The University of Dublin, Dublin, Ireland; National Yang-Ming University, TAIWAN

## Abstract

Asthma and chronic obstructive pulmonary disease (COPD) patients are required to inhale forcefully and deeply to receive medication when using a dry powder inhaler (DPI). There is a clinical need to objectively monitor the inhalation flow profile of DPIs in order to remotely monitor patient inhalation technique. Audio-based methods have been previously employed to accurately estimate flow parameters such as the peak inspiratory flow rate of inhalations, however, these methods required multiple calibration inhalation audio recordings. In this study, an audio-based method is presented that accurately estimates inhalation flow profile using only one calibration inhalation audio recording. Twenty healthy participants were asked to perform 15 inhalations through a placebo Ellipta™ DPI at a range of inspiratory flow rates. Inhalation flow signals were recorded using a pneumotachograph spirometer while inhalation audio signals were recorded simultaneously using the Inhaler Compliance Assessment device attached to the inhaler. The acoustic (amplitude) envelope was estimated from each inhalation audio signal. Using only one recording, linear and power law regression models were employed to determine which model best described the relationship between the inhalation acoustic envelope and flow signal. Each model was then employed to estimate the flow signals of the remaining 14 inhalation audio recordings. This process repeated until each of the 15 recordings were employed to calibrate single models while testing on the remaining 14 recordings. It was observed that power law models generated the highest average flow estimation accuracy across all participants (90.89±0.9% for power law models and 76.63±2.38% for linear models). The method also generated sufficient accuracy in estimating inhalation parameters such as peak inspiratory flow rate and inspiratory capacity within the presence of noise. Estimating inhaler inhalation flow profiles using audio based methods may be clinically beneficial for inhaler technique training and the remote monitoring of patient adherence.

## Introduction

Patients with chronic respiratory diseases such as asthma and chronic obstructive pulmonary disease (COPD) are instructed to perform a forceful, deep inhalation when using dry powder inhalers (DPIs) to ensure maximum dose delivery [1]. The peak inspiratory flow rate (PIFR) and volume or inspiratory capacity (IC) of the inhalation are critical in ensuring maximum clinical effectiveness from DPIs [2, 3]. It was reported in a study that over 20% of asthma patients could not generate a sufficient forceful inhalation with a DPI [4]. Furthermore, patients with advanced COPD generate less forceful inhalations with lower PIFRs [5]. There is a clinical need to remotely monitor patient inhalation technique during inhaler use so that the efficacy of inhaler medication is not reduced.

There is a lack of objective methods to monitor inhalation technique in inhalers, specifically DPIs, outside of the clinical environment. The In-Check Dial™ from Clement Clarke [Clement Clarke International Ltd, Harlow, UK] is a device used to measure patients’ PIFR through different simulated resistances that model different inhalers [5]. However, this cannot be used to remotely monitor inhalation technique in real DPIs. Audio-based methods have been employed to remotely monitor inhaler inhalation technique in DPIs and pMDIs [3, 6, 7]. The Inhaler Compliance Assessment (INCA) device is a non-invasive audio recording device that attaches to a Diskus™ inhaler and has been reported to be accurate at objectively measuring inhalation technique, specifically PIFR and IC in patients with asthma and COPD [8–10]. However, the flow-sound models employed to estimate PIFR and IC from inhalation audio recordings in these studies required numerous inhalation recordings from a cohort of participants. There is a need to introduce a faster, more efficient method of calibrating flow-sound models to accurately estimate not just PIFR and IC, but the entire flow profile of inhaler inhalations. Estimating the entire flow profile would allow healthcare professionals to monitor if patients can maintain the required flow rate throughout the inhalation.

Furthermore, changes in patient inhalation flow profiles may relate to physiological changes in respiratory conditions over time. It was previously reported that COPD patients tend to have poorer inhalation profiles than asthma patients in terms of PIFR and IC and that inhalation profiles change according to disease severity [5, 11, 12]. It was also reported previously that inhaler PIFR in COPD patients decreases by an average of approximately 15–18%, depending on the inhaler resistance, during an acute phase of an exacerbation [13]. In a previous pilot study, it was reported that a decline in lung function during a bronchial challenge test caused a decrease in inhaler PIFR which can be objectively measured using audio-based methods [14]. Therefore, by employing this audio-based method of accurately estimating the inhalation flow profile, it may be employed to remotely monitor respiratory health longitudinally also and possibly predict exacerbations before they occur.

Respiratory flow estimation using audio-based methods has been most thoroughly researched using microphones placed over the chest wall and trachea [15–17]. It was previously reported that accurate flow estimation may be achieved from tracheal audio recordings using only one audio recording with a corresponding flow signal for calibration. Although the acoustic properties of chest wall and tracheal sounds vary greatly to inhaler inhalation sounds recorded from a non-contact microphone, accurately modelling the flow-sound relationship of inhaler inhalations based on one calibration recording has yet to be investigated. If it was possible to develop an accurate model of monitoring inhalation flow profile based on one calibration inhalation recording, it would have significant clinical impact by allowing healthcare professionals to remotely monitor patient inhalation technique.

The aim of this study was to develop an accurate audio-based flow estimation model to estimate the inhalation flow profile in the Ellipta™ DPI. The hypothesis was that it was possible to accurately estimate the inhalation flow profile based on only one calibration inhalation audio recording.

## Methods

### Participants

Twenty healthy participants were recruited for this study. Written consent was obtained from study participants. In order to test the proposed method on a wide range of inspiratory flow rates, it was deemed necessary to recruit participants with healthy lung function. Baseline spirometry according to ATS/ERS standards was performed on each participant [18]. This study was approved by the Hospital Ethics Committee at Beaumont Hospital, Dublin, Ireland.

### Inhaler audio recording setup

**Fig 1:**
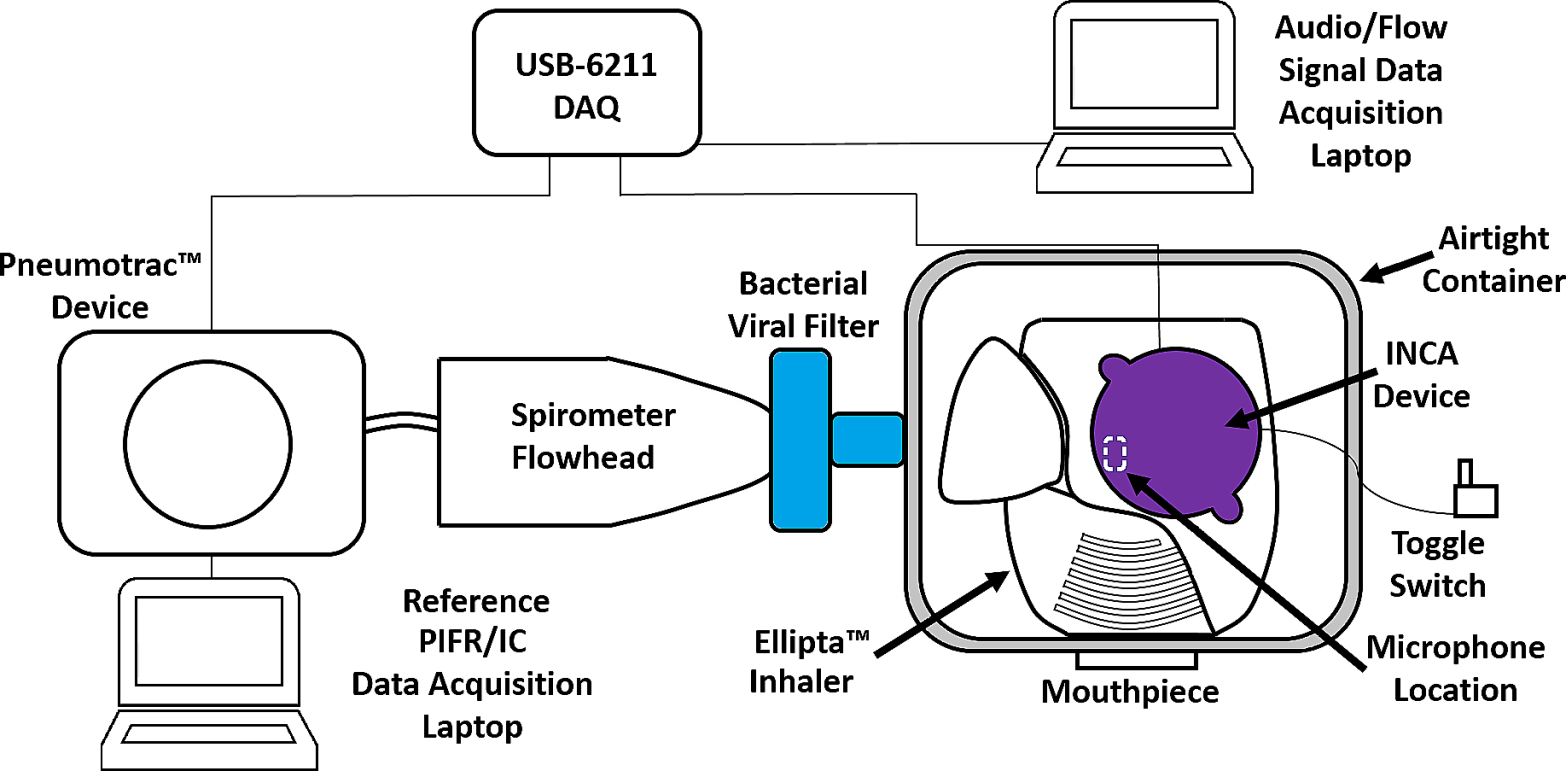
Inhaler recording setup.

Fig 1 shows the inhaler recording setup for this study. A placebo Ellipta™ DPI [GlaxoSmithKline, London, UK] was placed inside a custom built airtight container. An aperture was cut where a custom mouthpiece was inserted in order to give participants a better mouth seal when inhaling through the inhaler. An additional aperture was cut which allowed the INCA device to be placed directly onto the inhaler, as it would be in a clinical setting. This setup has been reported to give accurate audio-based measurements of flow rate in previous inhaler acoustics studies [3, 9]. A final aperture was cut to connect a Vitalograph Pneumotrac™ pneumotachograph spirometer [Vitalograph Ltd., Co. Clare, Ireland] to the airtight container. The spirometer was connected to a data acquisition laptop which allowed for reference measurements of PIFR and IC of inhalations using the Vitalograph Spirotrac® V software.

An audio signal was obtained directly from the INCA device and connected to a National Instruments USB-6211 DAQ system [National Instruments, Texas, USA]. The microphone used inside the INCA device is a Knowles SPU0414HR5H-SB microelectromechanical systems (MEMS) microphone [Knowles Acoustics, Illinois, USA]. The flow signal from the Pneumotrac™ pneumotachograph spirometer was obtained and connected to the DAQ also to record the inhalation flow profile signal. A custom designed LabVIEW Virtual Instrument [National Instruments, Texas, USA] was developed in order to record inhaler audio and flow signals simultaneously. Both audio and flow signals were sampled at 48 kHz and digitized at 16 bits per sample. Recordings took place in a designated recording office room. The recording room was not soundproof and so was not a completely noise-free environment. However, the recording environment was suitable to record inhalation audio signals with a sufficient signal to noise ratio to investigate the relationship between the audio and flow signals.

### Participant recording protocol

Each participant was instructed to exhale to functional residual capacity before inhaling forcefully through the mouthpiece of the Ellipta™ inhaler at maximal flow rate for as long as possible. This was then followed by a two to three second breath hold. This was repeated until five inhalation recordings were obtained at maximal inspiratory flow rate. All participants were able to inhale at 70 L/min or above at maximum effort. These five recordings were categorized as a High flow range group. A reference measurement of PIFR was checked using Vitalograph Spirotrac® V software to give participants feedback on their inspiratory flow rate after each inhalation recording. Participants were then asked to reduce their inspiratory flow rate for five recordings between 50–70 L/min (Medium flow range) and a final five recordings between 25–50 L/min (Low flow range). This gave a total of 15 inhalation recordings per participant.

### Audio and flow signal pre-processing

The DC component was removed from audio signals by subtracting the mean value of each signal from itself. Audio signals were high pass filtered with a cut off frequency of 200 Hz using a second order Butterworth filter to remove low frequency noise. Flow signals were low pass filtered with a cut off frequency of 4 Hz using a second order Butterworth filter. It was observed that a cut off frequency of 4 Hz could capture the rapid change in flow at the onset of inhalation while removing unwanted noise that did not represent the inspiratory flow. As the flow signals were originally recorded as voltage signals, the raw flow voltage (V) signals were converted to flow rate (L/min) using linear regression between the recorded reference PIFR on the Spirotrac® V software and the peak voltage in the flow signal over 20 recordings for each participant (15 recordings used in the audio analyses and an additional five training recordings used for flow signal calibration). This was calibrated for each participant. The average *R*^*2*^ value for converting the flow voltage signal to flow rate was 0.95±0.03 (± standard error) (p<0.0001) across all participants.

As this study only focused on the inhalation event, all exhalations and other respiratory sounds such as coughs were discarded in the audio and flow signals. Inhalations were segmented by determining the onset and offset points of the inhalation in the flow signal audio signal. A threshold of 5 L/min was chosen to segment inhalations to remove baseline noise being included in the flow signal. If the Virtual Instrument started recording data after the onset of an inhalation or if recording ceased towards the end of the inhalation, then the onset and offset thresholds were adjusted accordingly. However, this was only necessary in few recordings (7% of recordings).

### Audio feature extraction

An estimation of the acoustic envelope (amplitude envelope of the audio signal) of each inhaler inhalation was obtained using the Hilbert Transform (HT). This method has been employed in previous acoustical flow estimation studies [19, 20]. The estimate of the acoustic envelope, *x*_*env*_, was computed as the absolute value of the analytic signal, *x*_*a*_, which is a complex signal consisting of a summation of the inhalation audio signal with a 90° phase shifted version of itself (HT) [21]. The analytic signal is given by

$$x_{a}=x+j\hat{x}$$ (1)

Where *x* is the original inhaler inhalation audio signal and $\hat{x}$ is the HT of the original signal.

**Fig 2:**
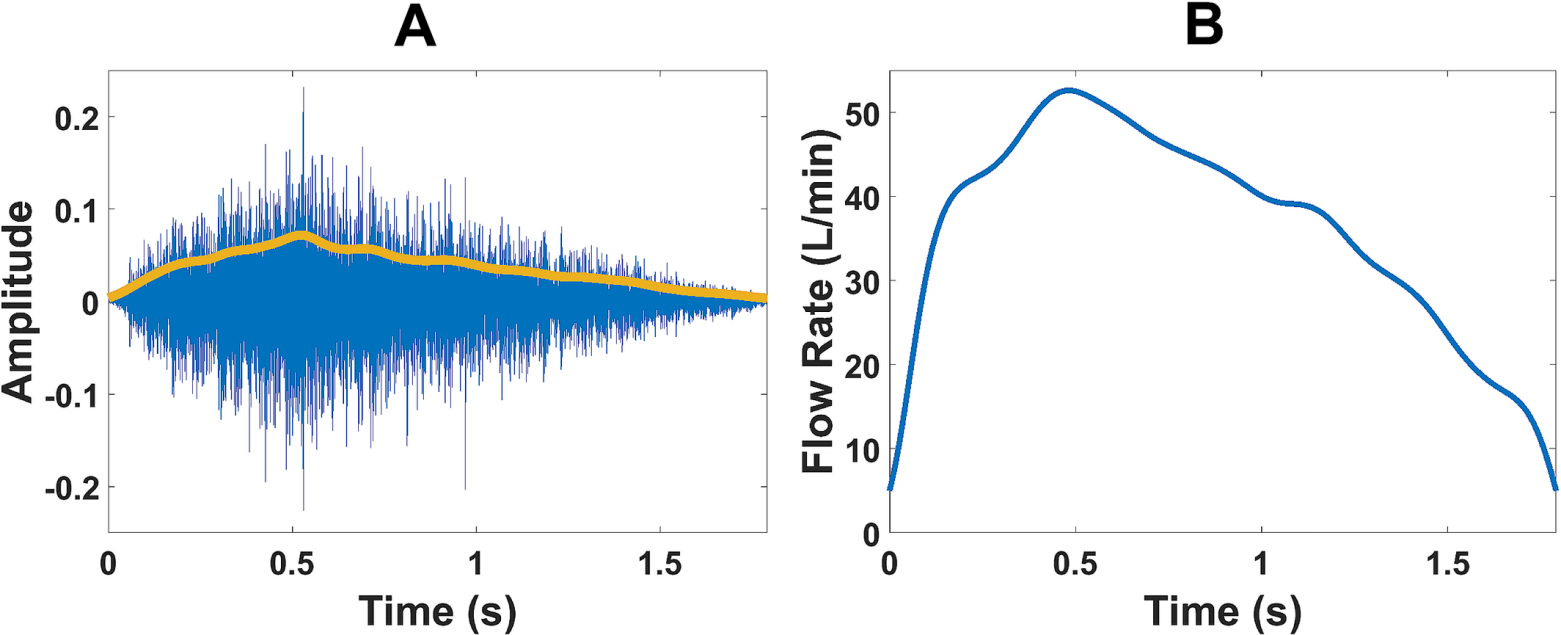
Example of inhaler inhalation audio and flow signals. (A) inhaler inhalation audio signal with estimated acoustic envelope and (B) corresponding flow signal.

The HT is usually most effective when applied to amplitude modulated narrow band signals. However, inhaler inhalation sounds recorded from a non-contact microphone (non-contact meaning not in contact or attached to the user) are composed of a broad spectrum of frequencies [6, 7]. Consequently, the absolute value of *x*_*a*_ follows high frequency changes in amplitude that distort the envelope estimation. The absolute value of the analytic signal, *x*_*a*_, was therefore low pass filtered with a cut off frequency of 4 Hz similar to the flow signal using a second order Butterworth filter. An example of the inhalation audio signal with the estimated acoustic envelope along with the corresponding flow signal after low pass filtering is presented in Fig 2. Data available from Dryad Digital Repository (https://doi.org/10.5061/dryad.8310n).

### Flow-sound regression model

Previous studies have reported the use of linear models to estimate flow rate using audio-based features [3, 6]. Other studies have reported that amplitude of sound and flow rate are related through a power law model [15, 22]. In order to investigate the relationship between the inhalation acoustic envelope and its corresponding flow signal, both linear and power law regression models were employed. A power law model may be calculated as a linear regression model in a logarithmic scale. The linear and power law regression models employed can be represented as

$$F_{est}=y∙x_{env}+z$$ (2)

$$\log\left(F_{est}\right)=a∙\log\;\left(x_{env}\right)+b$$ (3)

where *F*_*est*_ is the estimated flow signal and *x*_*env*_ is the acoustic envelope of the inhaler inhalation.

For each participant, one recording was used to calibrate a flow-sound regression model by computing the model coefficients (*y* and *z* for linear, *a* and *b* for power law) using the selected flow and audio signals. This model was then tested on the remaining 14 recordings for this participant to estimate the flow profiles from the remaining 14 audio signals. This process was repeated until each inhalation recording was used to calibrate a separate flow-sound regression model. In this way, a range of flow rates could be employed as single calibration recordings and then tested on a range of inspiratory flow rates to model real life patient inhaler use. This resulted in 210 tests (14 x 15 tests) being performed for each participant. Any data used to calibrate a model was not subsequently used to test the same resulting model as this may have introduced an over fitting bias. Once *F*_*est*_ was calculated using the model coefficients, a moving average window of 200 ms was also applied to the estimated flow signal from the time point at which 80% of PIFR was reached onwards to further remove noise from the estimated flow signal. The absolute average flow estimation error (absolute error between actual flow profile and estimated flow profile) was computed at each point and was then averaged. The average flow estimation error (*Average*_*error*_) over the entire inhalation signal was calculated using the following equation;

$$Average_{error}\;(\%)=\frac{1}{N}\sum_{i=1}^{N}\frac{\left|F_{est}(i)-F_{act}(i)\right|}{F_{act}(i)}\times 100$$ (4)

Where *F*_*est*_ is the estimated flow rate at the *i*^*th*^ point of the signal

*F*_*act*_ is the actual flow rate at the *i*^*th*^ point of the signal

*N* is the length of the actual and estimated flow signals.

Flow parameters that were calculated from the flow profile included PIFR, IC and the inhalation ramp time (Tr) which was pre-defined as the time taken to reach 80% of PIFR. PIFR was calculated as the peak flow point of the inhalation flow profile curve. The IC parameter was calculated as the area under the inhalation flow profile curve which equates to the total volume of air inhaled in liters. This was calculated using trapezoidal numerical integration of the flow and estimated flow signals. The Tr was calculated as the time at which the flow profile reached 80% of its maximum flow rate rather than the time taken to reach PIFR to avoid erroneous measurement due to small deviations when the flow profile plateaued.

Eq 5 details how the PIFR flow parameter estimation error was calculated. The IC and Tr estimation errors were also calculated using the same method accordingly.

$$PIFR_{error}\;(\%)=\frac{\left|PIFR_{est}-PIFR_{act}\right|}{PIFR_{act}}\times 100$$ (5)

All analyses were performed within participants and then results were averaged across participants.

In order to determine if the model coefficients remained unchanged according to calibration flow rate, a 2-sample t-test was performed to statistically compare regression model coefficients across inhalation flow ranges. These statistical tests were corrected for multiple comparisons using Bonferroni correction.

### Effect of noise on flow estimation

As inhalers are used by patients in different environments (both clinical and domestic), it is important to investigate the influence of noise on audio-based flow estimation. In order to investigate the effect of noise on this method of estimating the inhalation flow profile, Gaussian white noise was added to each inhalation audio signal. The method was tested on all audio signals at signal to noise ratio (SNR) levels of 0 dB, 5 dB, 10 dB, 15 dB, 20 dB and 25 dB. The accuracy of each parameter (Average, PIFR, IC and T_r_) was then calculated at each SNR level and averaged across all flow ranges and then averaged across all participants.

## Results

Participant information and baseline lung function is presented in Table 1. A total of 300 Ellipta™ inhaler inhalation recordings were obtained from 20 participants in this study. Thirteen recordings were discarded due to corrupt audio and flow signals leaving 287 recordings for analysis with 3,856 flow estimation tests being performed. Table 2 presents the average flow parameter values across the different flow ranges. This shows that the method was tested on a wide range of PIFR, IC and Tr values. The average flow parameters for each participant are presented in S1, S2 and S3 Tables.

**Table 1:** Participant information and baseline lung function (mean ± standard deviation).

Parameter	Value
Age (years)	24±2.5 (20–29)
Gender (M/F)	10/10
Height (cm)	171.95±8.75 (158–190)
Weight (kg)	67±13.18 (48–95)
BMI^a^ (kg/m^2^)	21.39±2.12 (19.2–25.9)
FEV1^b^ (L)	3.75±0.67 (2.79–5.12)
FEV1 Predicted %	97.65±13.04 (80–127)
FVC^c^ (L)	4.68±0.95 (3.38–6.73)
FEV1/FVC Ratio	0.82±0.06 (0.72–0.91)
PIFR^d^ (L/min)	329.35±93.3 (193–485)

^a^ BMI–body mass index

^b^ FEV1 –forced expiratory volume in one second

^c^ FVC–forced vital capacity

^d^ PIFR–peak inspiratory flow (during spirometry, not inhaler usage)

**Table 2:** Mean (± standard deviation) of flow parameter values from recorded inhalation flow signals averaged across all participants.

Flow Range	PIFR (L/min)	IC (L)	Tr (ms)
High (≥70 L/min)	109±21 (70–142)	2.81±0.7 (1.82–4.09)	317±145 (134–597)
Medium (50–70 L/min)	60±5 (52–66)	1.77±0.5 (1.18–2.98)	317±136 (155–770)
Low (25–50 L/min)	39±4 (32–44)	1.33±0.5 (0.71–2.48)	323±107 (146–562)

**Fig 3:**
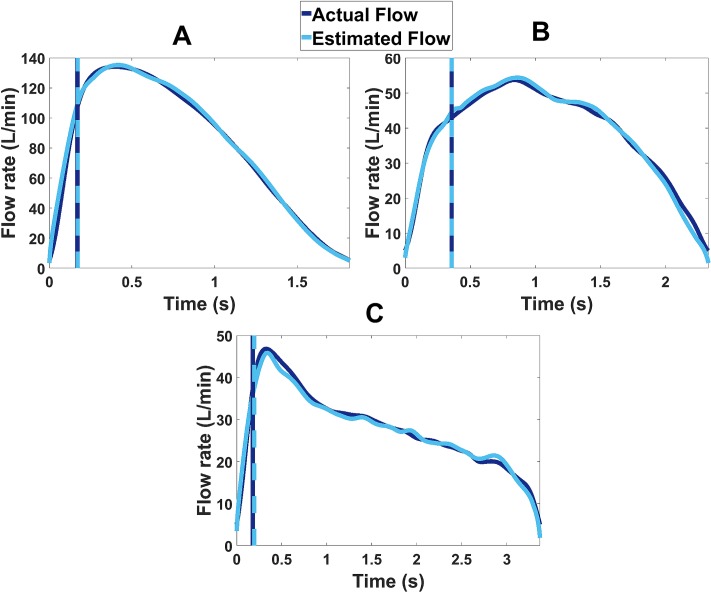
Examples of actual and estimated inhaler inhalation flow profiles. (A) High, (B) Medium and (C) Low flow rates. The vertical lines represent the actual and estimated Tr values.

It was observed that the power law models were superior over the linear models at estimating the inhalation flow profile using the acoustic envelope. The average flow estimation accuracy (which was calculated as 100-*Average*_*error*_) was 90.89±0.9% (± standard error) for power law and 76.63±2.38% for linear models. Therefore, detailed results of the power law models are discussed from this point onwards in the study. Average flow parameter estimation errors were divided according to calibration and test flow ranges. The overall *Average*_*error*_ for the power law models was 9.4±0.92% for High flow range, 8.65±1.67% for Medium flow range and 9.29±2.44% for Low flow range (± standard error). This gave an average estimation error of 9.11±0.9% across all participants. Fig 3 presents examples of the estimated flow signals for high, medium and low flow inhalations.

**Fig 4:**
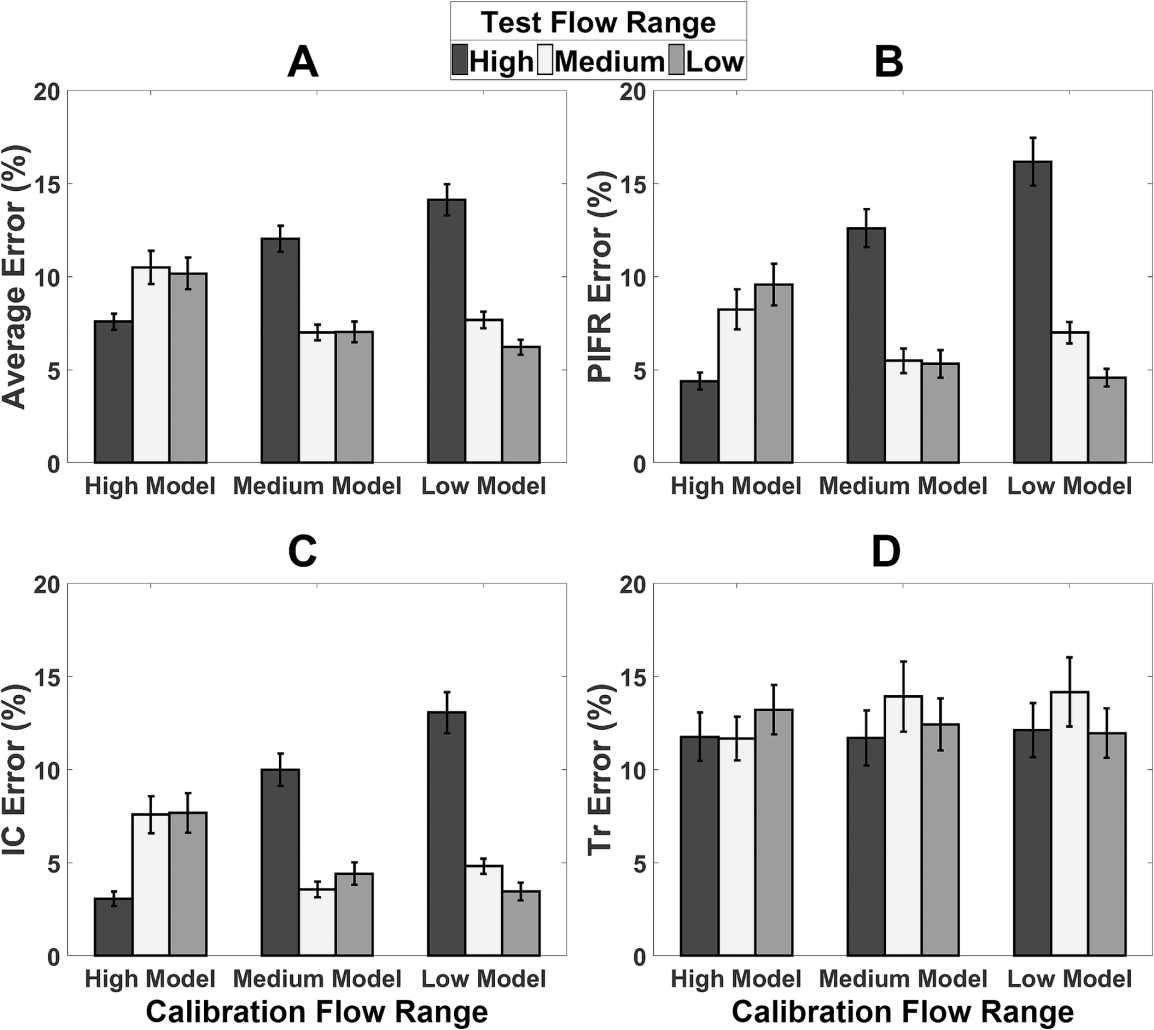
Average audio-based flow estimation errors. (A) *Average*_*error*_, (B) *PIFR*_*error*_, (C) *IC*_*error*_ and (D) *Tr*_*error*_ (%) (± standard error). Results are divided into models calibrated using high, medium and low flow inhalation recordings and tested on high, medium and low flow ranges.

Fig 4 shows the *Average*_*error*_, *PIFR*_*error*_, *IC*_*error*_ and *Tr*_*error*_ values averaged across all participants. The average flow estimation accuracy for each participant across all flow ranges is presented in S1 Fig.

The acoustic envelope of the inhalation audio signal was strongly significantly linearly correlated with the flow signal in a logarithmic scale. The average (± standard error) *R*^*2*^ values of the flow-sound regression models created during calibration were 0.9791±0.0027 for High flow range, 0.98±0.0023 for Medium flow range and 0.9778±0.0019 for Low flow range (p<0.0001). Although this is not a measurement of flow estimation accuracy, it is important to note the statistical correlation between the audio envelope and the flow signal.

**Fig 5:**
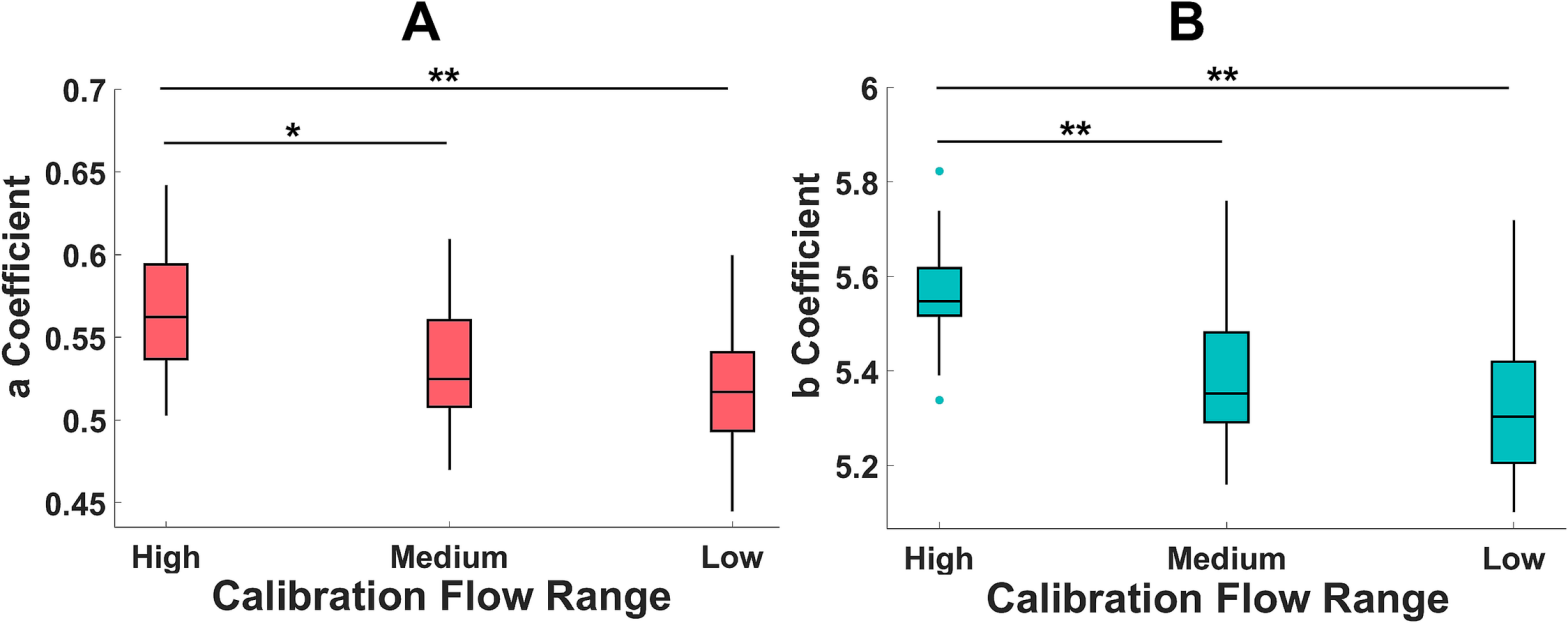
Boxplots representing the median, interquartile range and 1.5×interquartile range of regression coefficients. (A) *a* and (B) *b* regression coefficients where *p<0.05 and **p<0.01 corrected for multiple comparisons using Bonferroni correction.

It was observed that the *a* and *b* model coefficients at the High flow range were statistically significantly higher than those in the Medium (p<0.05 for *a* coefficient, p<0.01 for *b* coefficient) and Low (p<0.01 for both *a* and *b* coefficients) flow ranges. This suggests that the relationship between the flow and acoustic envelope may not be constant at all calibration flow rates. Fig 5 shows boxplots of the model coefficients across all participants in the High, Medium and Low flow ranges.

**Fig 6:**
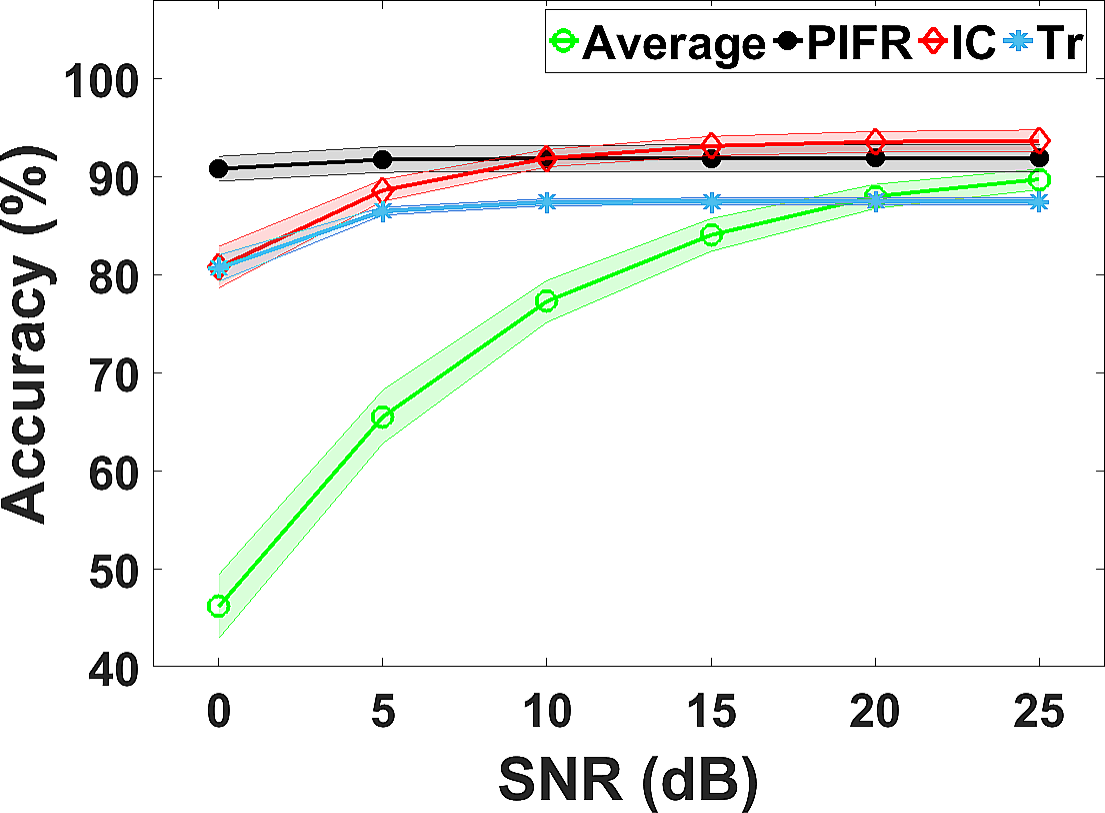
Average accuracy (%) of inhalation flow profile parameter estimation across all flow rates at different SNR levels.

Fig 6 presents the average accuracy of estimating different inhalation parameters at a range of SNR levels (0–25 dB in increments of 5dB). It can be observed from Fig 6 that PIFR, IC and T_r_ achieve high accuracy above 80% even at very low SNR levels. The Average flow estimation accuracy decreases with lower SNR levels but remains above 70% at 10 dB.

## Discussion

It was observed that the acoustic envelope of the inhalation audio signal and its corresponding flow signal followed a power law relationship which can be estimated as a linear model in a logarithmic scale. This agrees with previous studies that employed power and amplitude based features to estimate respiratory flow [15, 22]. Power law models generated an average flow estimation accuracy of over 10% higher than linear models (90.89% for power law models vs. 76.63% for linear models). By estimating the acoustic envelope of the inhalation audio signal, it was possible to accurately estimate the inhaler inhalation flow profile. This may allow healthcare professionals to objectively assess patient inhalation technique and medication adherence in future clinical applications.

When the calibration and test recordings were from the same flow range, this generated the smallest error which would be expected. The largest error in estimating flow parameters was observed when the calibration recording was obtained from a low flow inhalation and was used to estimate a high flow inhalation. This would also be expected as one can see from Fig 5 that the model coefficients change at higher flow rates compared to lower flow rates. Therefore, the relationship between flow and sound may not be constant at all flow rates. Furthermore, the difference in model coefficients across flow ranges did not greatly impair flow estimation in inhaler inhalations in this study. The High flow range had much higher flow rates than the Medium and Low flow ranges. It was noted that the T_r_ values were not significantly different across flow range groups also. This may suggest that the acceleration of inhalations may have influenced the model coefficients also. Therefore, it could be argued that the model coefficients change most significantly at high flow rates with faster acceleration.

There were some limitations to this study. Healthy participants were recruited in order to investigate the accuracy of this method on a wide range of flow rates. This may not have been possible with patients suffering from impaired respiratory function. In addition, asking patients to inhale at a range of different flow rates may confuse patients on how to correctly use a DPI. Consequently, patients were not recruited for this study, however, the efficacy of this audio-based method in estimating inhalation flow profiles has been presented. The airtight container may have affected inhaler inhalation audio-based features, particularly spectral features. However, recording directly from the surface of the inhaler and not inside the airtight container, as well as using an energy based feature such as the acoustic envelope would minimize this effect. The airtight container also ensured accurate measurement of flow rate without the need for sensors within the inhaler device. Flow sensors placed within the inhaler may have changed the resistance within the inhaler and would affect flow measurement.

Recordings took place in a designated recording room to reduce background noise in the audio recordings which may not replicate real life clinical environments. In order to fully understand the relationship between audio and flow signals, it was essential to reduce background noise. The microphone in the INCA device was situated very close (approximately 2 cm) to the grill at the Ellipta™ mouthpiece where the inhalation sound is mostly generated. Furthermore, the SNR was high (13.9–23 dB) in low to high flow inhalations even with ambient background noise present in the recording room. Furthermore, this method was tested on a range of SNR levels and generated sufficient accuracy in estimating parameters such as PIFR IC and T_r_ even within lower SNR levels. This shows the robustness of this flow estimation method in noisy environments. As would be expected, the average flow estimation accuracy decreased at lower SNR levels. However, as the microphone is positioned within the device casing directly beside the inhaler mouthpiece, this should generate sufficient SNR levels even in real clinical environments. Future research will test this method in clinical and domestic environments.

Future research should also focus on the automatic detection of Ellipta™ inhalation sounds from noisy environments. Once the inhalation sounds are accurately detected, one can apply the methods presented in this study to accurately assess patient inhaler inhalation technique. The flow signal was used in this study to segment inhalations to investigate the relationship between the audio and flow signals. Previous studies by Holmes et al developed an accurate method of detecting patient inhalation sounds recorded from the INCA device attached to a Diskus DPI during inhaler use in real clinical and domestic environments [23, 24]. However, there is a clinical need for further audio-based classification methods to accurately detect inhalations from Ellipta™ DPI as well as testing the accuracy of the presented method on estimating low, medium and high inspiratory flow rates within noisy real-life environments.

The presented audio-based flow estimation method could be implemented into the clinical setting by obtaining one recording of an inhaler inhalation during a patient’s consultation with a healthcare professional. As the patient is trained on their inhaler, an inhalation could be recorded using INCA and used to calibrate a model to estimate the inhaler inhalation flow profile remotely. When the patient returns for a consultation, the patient may obtain objective feedback on their inhalation technique and can be trained on how to improve their inhaler technique and their disease control as a result. Monitoring inhalation flow parameters remotely using acoustics provides a non-invasive, accurate method of monitoring inhaler user technique and potentially the respiratory health of asthma and COPD patients.

## Conclusions

This study presented a method of accurately estimating the flow profile of inhaler inhalations based on the logarithmic relationship between the acoustic envelope of the inhalation sound and flow signal. Using only one inhalation recording for model calibration, an average flow estimation accuracy of over 90% was observed. This method may be employed to remotely monitor patient inhalation technique and help train patients to improve their inhaler technique by introducing more personalized treatments in respiratory medicine. Future research will investigate employing audio-based classification methods to segment inhalation sounds in noisy clinical and domestic environments. Other future research will apply the presented flow estimation method to other DPIs to remotely monitor patient adherence across a range of different inhaler devices.

## Supporting information

S1 Table Average PIFR values from inhalation flow profiles for each participant. (DOCX)

S2 Table Average IC values from inhalation flow profiles for each participant. (DOCX)

S3 Table Average Tr values from inhalation flow profiles for each participant. (DOCX)

S1 Fig Average flow estimation accuracy (± standard error) averaged across all flow ranges for each participant. (DOCX)
