# Supplementary material for: Estimation of inhalation flow profile using audio-based methods to assess inhaler medication adherence
Source: PLoS One. 2018 Jan 18;13(1):e0191330. doi: 10.1371/journal.pone.0191330 (PMC5773205; doi:10.1371/journal.pone.0191330)
Supplement: S1 Table — (DOCX) [file pone.0191330.s001.docx]

S1 Table. Average PIFR values from inhalation flow profiles for each participant.

| **Participant No.** | **PIFR – High (L/min)** | **PIFR – Medium (L/min)** | **PIFR – Low (L/min)** |
| --- | --- | --- | --- |
| 1 | 79 | 64 | 42 |
| 2 | 107 | 66 | 42 |
| 3 | 132 | 54 | 44 |
| 4 | 134 | 60 | 33 |
| 5 | 102 | 66 | 43 |
| 6 | 97 | 58 | 34 |
| 7 | 134 | 53 | 35 |
| 8 | 100 | 62 | 43 |
| 9 | 70 | 55 | 42 |
| 10 | 123 | 52 | 32 |
| 11 | 113 | 58 | 37 |
| 12 | 115 | 61 | 40 |
| 13 | 142 | 61 | 38 |
| 14 | 129 | 64 | 42 |
| 15 | 126 | 65 | 41 |
| 16 | 83 | 63 | 38 |
| 17 | 87 | 54 | 41 |
| 18 | 88 | 60 | 42 |
| 19 | 112 | 62 | 35 |
| 20 | N/A | 57 | 41 |
