# Supplementary material for: Estimation of inhalation flow profile using audio-based methods to assess inhaler medication adherence
Source: PLoS One. 2018 Jan 18;13(1):e0191330. doi: 10.1371/journal.pone.0191330 (PMC5773205; doi:10.1371/journal.pone.0191330)
Supplement: S2 Table — (DOCX) [file pone.0191330.s002.docx]

S2 Table. Average IC values from inhalation flow profiles for each participant.

| **Participant No.** | **IC – High Flow (L)** | **IC – Medium Flow (L)** | **IC – Low Flow (L)** |
| --- | --- | --- | --- |
| 1 | 1.90 | 1.28 | 0.94 |
| 2 | 2.33 | 1.19 | 0.96 |
| 3 | 2.86 | 1.98 | 2.07 |
| 4 | 3.09 | 1.31 | 0.84 |
| 5 | 2.61 | 1.73 | 1.40 |
| 6 | 2.64 | 1.61 | 1.01 |
| 7 | 2.53 | 1.57 | 1.11 |
| 8 | 2.16 | 1.88 | 1.48 |
| 9 | 2.68 | 1.98 | 1.45 |
| 10 | 3.95 | 2.98 | 2.48 |
| 11 | 2.36 | 1.20 | 0.92 |
| 12 | 3.73 | 2.07 | 1.45 |
| 13 | 4.02 | 1.18 | 0.71 |
| 14 | 3.27 | 2.03 | 1.28 |
| 15 | 4.09 | 2.84 | 2.28 |
| 16 | 1.82 | 1.25 | 0.95 |
| 17 | 2.47 | 1.75 | 1.80 |
| 18 | 2.36 | 1.81 | 0.87 |
| 19 | 2.57 | 1.88 | 1.20 |
| 20 | NA | 1.84 | 1.45 |
