# Supplementary material for: Estimation of inhalation flow profile using audio-based methods to assess inhaler medication adherence
Source: PLoS One. 2018 Jan 18;13(1):e0191330. doi: 10.1371/journal.pone.0191330 (PMC5773205; doi:10.1371/journal.pone.0191330)
Supplement: S3 Table — (DOCX) [file pone.0191330.s003.docx]

S3 Table. Average Tr values from inhalation flow profiles for each participant.

| **Participant No.** | **Tr – High Flow (ms)** | **Tr – Medium Flow (ms)** | **Tr – Low Flow (ms)** |
| --- | --- | --- | --- |
| 1 | 304 | 306 | 390 |
| 2 | 223 | 155 | 146 |
| 3 | 134 | 251 | 240 |
| 4 | 188 | 175 | 245 |
| 5 | 409 | 344 | 336 |
| 6 | 563 | 770 | 464 |
| 7 | 168 | 244 | 317 |
| 8 | 329 | 314 | 214 |
| 9 | 485 | 239 | 241 |
| 10 | 335 | 470 | 390 |
| 11 | 270 | 195 | 171 |
| 12 | 232 | 373 | 280 |
| 13 | 164 | 176 | 341 |
| 14 | 254 | 299 | 243 |
| 15 | 315 | 376 | 562 |
| 16 | 577 | 347 | 436 |
| 17 | 193 | 258 | 302 |
| 18 | 597 | 361 | 400 |
| 19 | 286 | 407 | 448 |
| 20 | NA | 281 | 285 |
