# Supplementary material for: Estimation of inhalation flow profile using audio-based methods to assess inhaler medication adherence
Source: PLoS One. 2018 Jan 18;13(1):e0191330. doi: 10.1371/journal.pone.0191330 (PMC5773205; doi:10.1371/journal.pone.0191330)
Supplement: S1 Fig — (DOCX) [file pone.0191330.s004.docx]

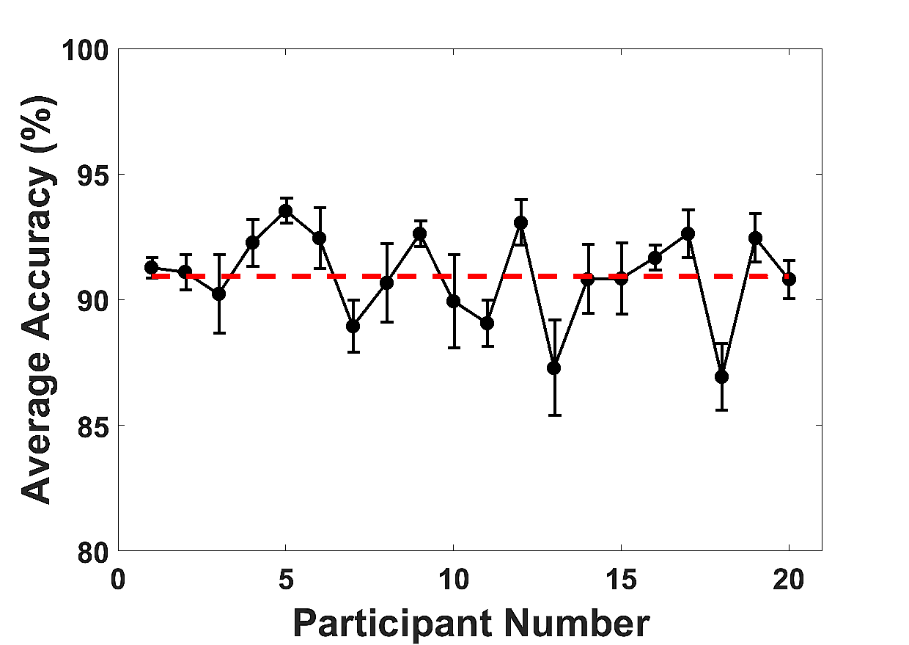


S1 Fig. Average flow estimation accuracy (± standard error) averaged across all flow ranges for each participant.
